# Immune regulatory mechanisms of M2 macrophage polarization and efferocytosis in diabetic kidney disease: an integrated screening study with therapeutic implications

**DOI:** 10.3389/fendo.2025.1652402

**Published:** 2025-10-06

**Authors:** Yi Kang, Qian Jin, Mengqi Zhou, Huijuan Zheng, Danwen Li, Xuezhe Wang, Jingwei Zhou, Jie Lv, Yaoxian Wang

**Affiliations:** ^1^ Dongzhimen Hospital, Beijing University of Chinese Medicine, Beijing, China; ^2^ Renal Research Institution, Beijing University of Chinese Medicine, Beijing, China; ^3^ Graduate School, Beijing University of Chinese Medicine, Beijing, China; ^4^ Department of Traditional Chinese Medicine, Beijing Puren Hospital, Beijing, China

**Keywords:** diabetic kidney disease, M2 macrophage, efferocytosis, bioinformatics, immune regulatory

## Abstract

**Background:**

The imbalance in macrophage phenotype transition is a central mechanism driving chronic inflammation in diabetic kidney disease (DKD). Macrophages can polarize toward the M2 phenotype via efferocytosis, exerting anti-inflammatory and pro-resolving effects. However, the identification and functional validation of regulatory genes governing M2 macrophage and efferocytosis in DKD remain to be thoroughly explored.

**Methods:**

Differentially expressed genes were obtained based on GSE96804 and GSE30122 data sets. Based on efferocytosis-related genes (ERGs) and M2 polarization-related genes (MRGs), ERG and MRG scores were computed in the GSE96804 dataset. Weighted gene co-expression network analysis (WGCNA) was carried out to identify critical module genes. Finally, macrophage-efferocytosis-related DEGs (MEDEGs) were identified. Further, machine learning (ML)—support vector machine (SVM), BORUTA, and lasso regression—were employed to identify hub genes and build Nomogram predictive model. Additionally, hub genes were confirmed through animal experiments.

**Results:**

A total of 35 MEDEGs were identified. ML recognized 3 hub genes—MCUR1, CYP27B1, and G6PC. Hub genes were notably downregulated in DKD group and exhibited high predictive ability. Furthermore, the Nomogram model based on key genes has shown potential in predicting DKD. The findings were further validated through transcriptome sequencing of DKD model.

**Conclusion:**

This study uncovered 3 hub genes—MCUR1, CYP27B1, and G6PC—linked to M2 polarization, efferocytosis, and DKD. These genes may contribute to DKD pathogenesis, providing novel targets for early diagnosis and therapeutic interventions in DKD.

## 1 Introduction

Diabetic kidney disease (DKD) is one of the most severe microvascular complications of diabetes (1). According to the Global Burden of Disease study, DKD accounts for more than 50% of new dialysis cases in developed countries (2). Despite advancements in glycemic control and renin-angiotensin system blockade therapies, 30-40% of patients still progress to irreversible renal failure (3). This clinical challenge highlights the urgent need to elucidate novel pathogenic mechanisms. The kidney is a complex organ composed of more than 50 different cell types (4).

The development of kidney diseases is driven by complex intercellular interactions in the renal microenvironment (5). Previous DKD studies have primarily focused on hyperglycemia-induced direct injury mechanisms affecting renal (6–8). However, the underlying pathogenic mechanisms remain unclear, and there is a lack of sensitive and efficient predictive biomarkers. Once DKD progresses to overt nephropathy, it rapidly advances to the end-stage (9).

Recent findings suggest that chronic inflammation is a key factor driving DKD, with macrophage-mediated immune dysregulation playing a crucial role in sustained renal injury (10, 11). Macrophage-dominant immune cell infiltration is observed in renal tissues of DKD patients at various disease stages (12), and the extent of macrophage infiltration is significantly correlated with the rate of renal function decline in DKD patients (13). The pro-inflammatory M1 phenotype dominates the early stages of injury, whereas M2 macrophages contribute to anti-inflammatory responses and tissue repair. The imbalance in macrophage polarization is a crucial factor driving the persistent progression of inflammation (14). Efferocytosis is the process by which phagocytic innate immune cells clear apoptotic cells. It plays a pivotal role in maintaining immune homeostasis, controlling chronic inflammation, and facilitating tissue repair (15). Mechanistically, efferocytosis not only eliminates cellular debris but also promotes the polarization of macrophages toward the anti-inflammatory and reparative M2 phenotype, establishing a self-sustaining reparative loop (16). Macrophage efferocytosis is a critical step in inflammation resolution. Impairment in this process exacerbates tubular atrophy and glomerulosclerosis (17).

**Figure 1:**
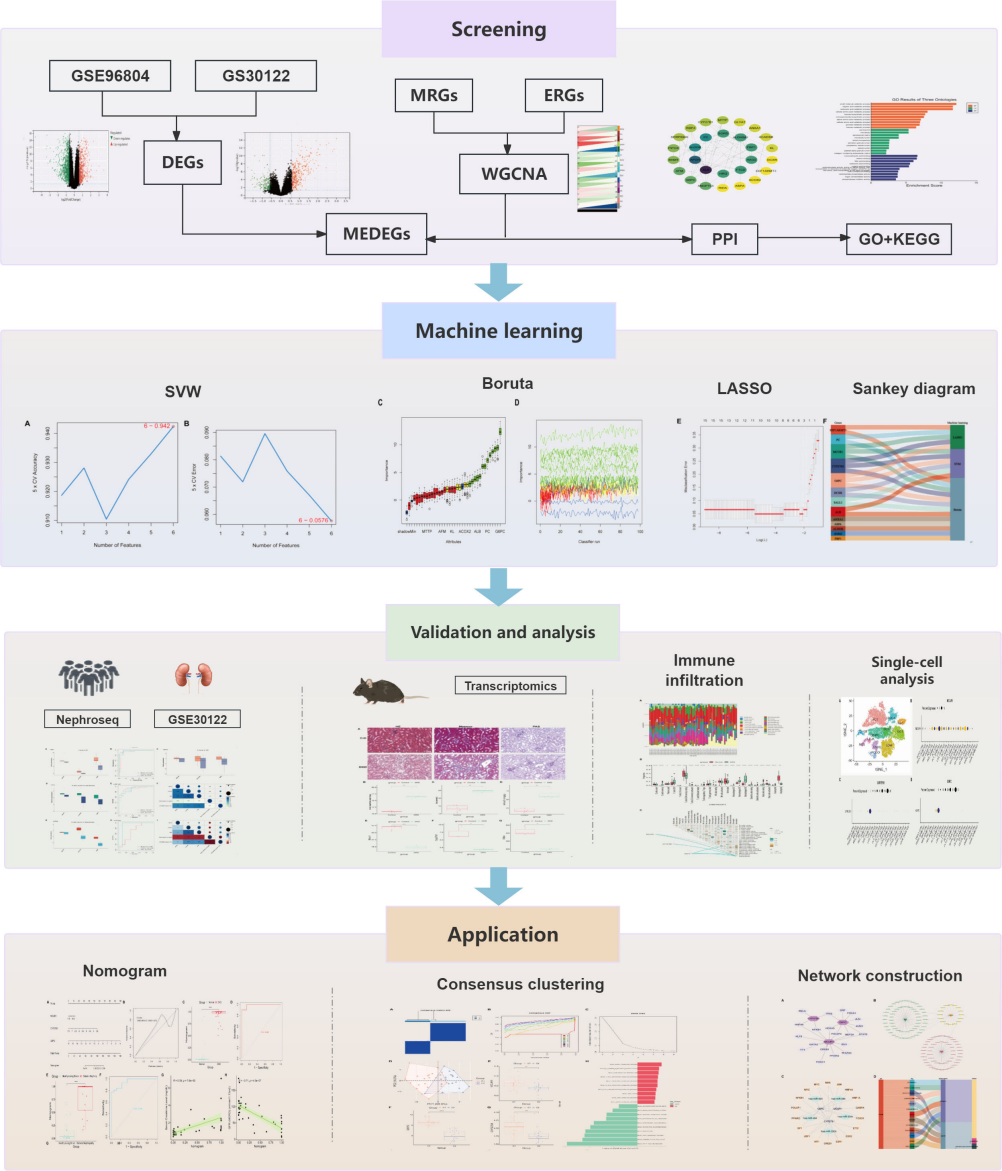
Research flowchart.

This study uses bioinformatics techniques to explore the potential role of M2 polarization and efferocytosis-associated genes. Machine learning (ML) algorithms are then applied to pinpoint biomarkers closely tied to DKD pathology (**Figure 1**).

## 2 Methods

### 2.1 Data sources

The DKD gene expression datasets GSE96804 and GSE30122 were retrieved from gene expression omnibus (GEO) database (**Table 1**). The keyword “efferocytosis” was used to perform a search in the GeneCards database (https://www.genecards.org/). Based on literature extraction, 137 efferocytosis-related genes (ERGs) were identified (18–20), and after integration, 137 ERGs were derived (**Supplementary Table S1**). Additionally, based on previous studies (21), we identified 46 genes associated with M2 macrophage polarization (MRGs) (**Supplementary Table S2**).

**Table 1:** Overview of the datasets.

Dataset	Database	Platform	Type
GSE96804	GEO	GPL17586	41 DKD cases and 20 normal case
GSE30122	GEO	GPL571	19 DKD cases and 50 normal case
Ju CKD Glom	Nephroseq	/	12 DKD cases and 21 normal case
Ju CKD TubInt	Nephroseq	/	17 DKD cases and 31 normal cases

### 2.2 Differentially expressed genes

The limma package (22) was employed to analyze the GSE96804 and GSE30122 datasets. DEGs from GSE96804 were designated as DEGs 1, while those from GSE30122 were labeled as DEGs 2. The selection criteria for DEGs were a fold change (FC) > 1.5 and adj.*P* < 0.05. Heatmaps (pheatmap package) and Volcano plots (ggplot2 package (23)) were used to visualize DEGs expression patterns. Finally, the overlapping DEGs genes from the two datasets were integrated to form the final DEGs.

### 2.3 Single sample gene set enrichment analysis scoring

We performed ssGSEA using the GSVA package (24), with MRGs and ERGs as the reference background set. ssGSEA enrichment scores were computed for all samples in the GSE96804 dataset.

### 2.4 Weighted gene co-expression network analysis analysis

WGCNA is a powerful tool for analyzing gene expression data, aiming to identify gene modules by constructing gene co-expression networks and further revealing key genes within these modules. Genes were filtered by calculating the median absolute deviation (MAD) and retaining the top 50% with highest variability. The WGCNA package’s goodSamplesGenes function was then applied to remove outliers (25). A scale-free co-expression network was subsequently built using WGCNA. MRG and ERG scores were considered as trait variables, and *pearson* correlation analysis was performed between modules and MRGs/ERGs scores.

### 2.5 Enrichment analysis

The intersection of DEGs and module genes was obtained to identify macrophage-efferocytosis related DEGs (MEDEGs). The STRING database (https://cn.string-db.org/) was utilized to construct the protein-protein interaction (PPI) network. Functional enrichment analysis of gene ontology (GO) terms and kyoto encyclopedia of genes and genomes (KEGG) pathways was performed with the clusterProfiler R package.

### 2.6 ML selection of hub genes

Three ML methods—lasso regression, support vector machine (SVM), and BORUTA algorithm—were employed to filter MEDEGs. Lasso regression enhances model interpretability through regularization (26). SVM is employed to manage high-dimensional data and optimize the classification boundary and the BORUTA algorithm identifies significant features by evaluating the relative importance of variables. The intersection was considered as hub genes.

### 2.7 Validation and clinical significance

The pROC package (27) was employed to calculate the area under the ROC curve (AUC) to evaluate predictive performance. Furthermore, the correlation between hub genes and clinical parameters such as serum creatinine (Scr) and glomerular filtration rate (GFR) was examined using the GSE30122 dataset and the Nephroseq database (http://v5.nephroseq.org). Furthermore, Nomogram was constructed using the rms package.

### 2.8 Immune infiltration analysis

The CIBERSORT algorithm was applied to assess immune infiltration levels. The “PERM” parameter was set to 100, with a critical value of *P* at 0.05. Assessing the correlation between hub genes and immune cells, and heatmap was generated to visualize associations.

### 2.9 Single-cell RNA sequencing analysis

Sc-RNA seq datas were analyzed using the Kidney Integrated Transcriptomics (K.I.T.) database (https://www.humphreyslab.com/SingleCell/). Wilson P et al. (28)conducted unbiased Sc-RNA seq on cryopreserved human diabetic kidney samples. This study extracted sc-RNA seq data of hub genes in DKD samples and visualized the results.

### 2.10 Consensus clustering

Consensus clustering analysis was conducted on hub genes expression matrix using the Consensus Cluster Plus package (29). The similarity between the samples was assessed using Euclidean distance, followed by K-means clustering. Subsequently, 500 iterations were performed with a resampling proportion of 0.8. Next, principal component analysis (PCA) was subsequently employed to validate the expression patterns of genes under different subtypes. Additionally, the gene set variation analysis (GSVA) expression profiles of different subtypes were compared.

### 2.11 Molecular regulatory networks and drug prediction

Transcription factors (TFs) were predicted through the NetworkAnalyst 3.0 platform (JASPAR database) (https://www.networkanalyst.ca/). The miRTarBase database was used to predict miRNA targets. Next, the mRNA-TF, mRNA-miRNA, and TF-miRNA-mRNA interaction networks were constructed to elucidate their regulatory roles. Furthermore, drug prediction for hub genes was conducted using the DGIdb database (https://www.dgidb.org/).

### 2.12 Animal model validation

All methods were carried out in accordance with relevant guidelines and regulations in the manuscript. All animal experimental procedures were in accordance with ARRIVE guidelines.

Twenty-four SPF-grade male C57BL/6J mice (20 ± 2 g, 6–8 weeks) were purchased from Beijing Vital River Laboratory Animal Technology Co., Ltd. (Production License No.: SCXK (Beijing)-20210006). This study was approved by the Animal Ethics Committee of Beijing University of Chinese Medicine (BUCM-2023120104-4282). The detailed experimental procedures are provided in the **Supplementary Materials**. Under 1% pentobarbital sodium anesthesia administered intraperitoneally (ip), the kidneys were completely excised, and the cortical tissue was divided into two parts: one was fixed in 4% paraformaldehyde for 24 hours for histopathological evaluation using HE, PAS, and Masson staining; the other was snap-frozen in liquid nitrogen and submitted to Shanghai Majorbio Bio-pharm Technology Co.,Ltd. for transcriptomic sequencing.

### 2.13 Statistical analysis

Statistical analyses were conducted using R Studio 4.3.0. *Pearson* correlation analysis was used to evaluate relationships between variables, and the Wilcoxon test was applied to compare differences between the two groups. *P*-value < 0.05 was considered statistically significant.

## 3 Results

### 3.1 DKD is associated with efferocytosis and M2 macrophage polarization

**Figure 2:**
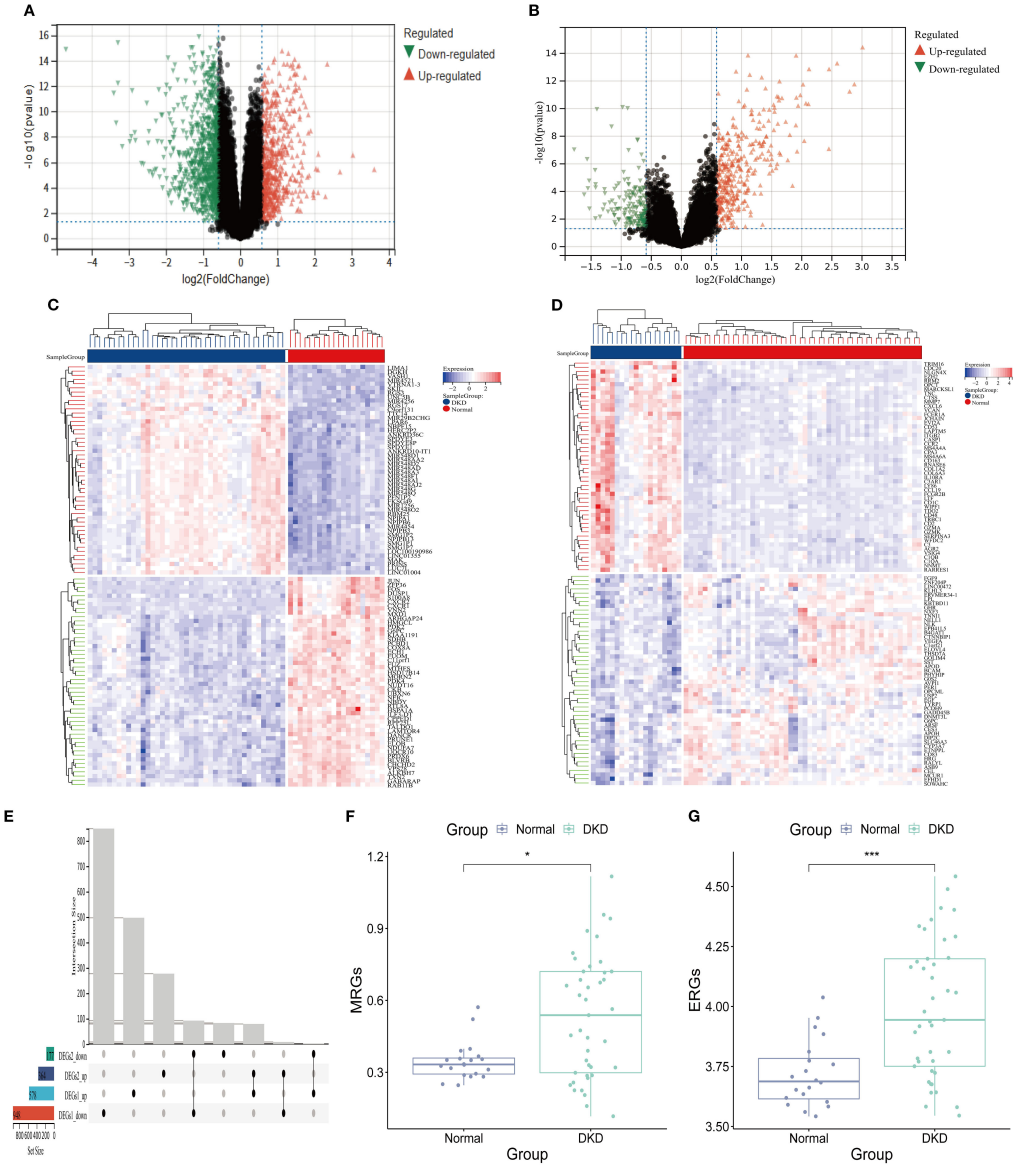
Efferocytosis and M2 macrophage polarization in DKD. **(A, B)** Volcano plot. **(A)** DEGs 1; **(B)** DEGs 2. **(C, D)** Heatmap. **(C)** DEGs 1; **(D)** DEGs 2; **(E)** Upset plot of intersecting DEGs. **(F)** MRGs scores **(G)** ERGs scores (^*^ *P* < 0.05,^****^ *P* < 0.001).

In the GSE96804 dataset, differential expression analysis identified 578 significantly upregulated genes (DEGs 1_up) and 948 significantly downregulated genes (DEGs 1_down) (**Figures 2A, C**; **Supplementary Table S3**). Similarly, in the GSE30122 dataset, 364 upregulated genes (DEGs2_up) and 177 downregulated genes (DEGs2_down) were detected (**Figures 2B, D**; **Supplementary Table S4**). Finally, 171 DEGs were obtained (**Figure 2E**). ssGSEA revealed a notable difference between the DKD and normal group (*P* < 0.001). Notably, the ssGSEA score was significantly elevated in DKD group (**Figures 2F, G**), suggesting that the pathological progression of DKD may involve the regulation of M2 macrophage polarization and efferocytosis.

### 3.2 WGCNA analysis

**Figure 3:**
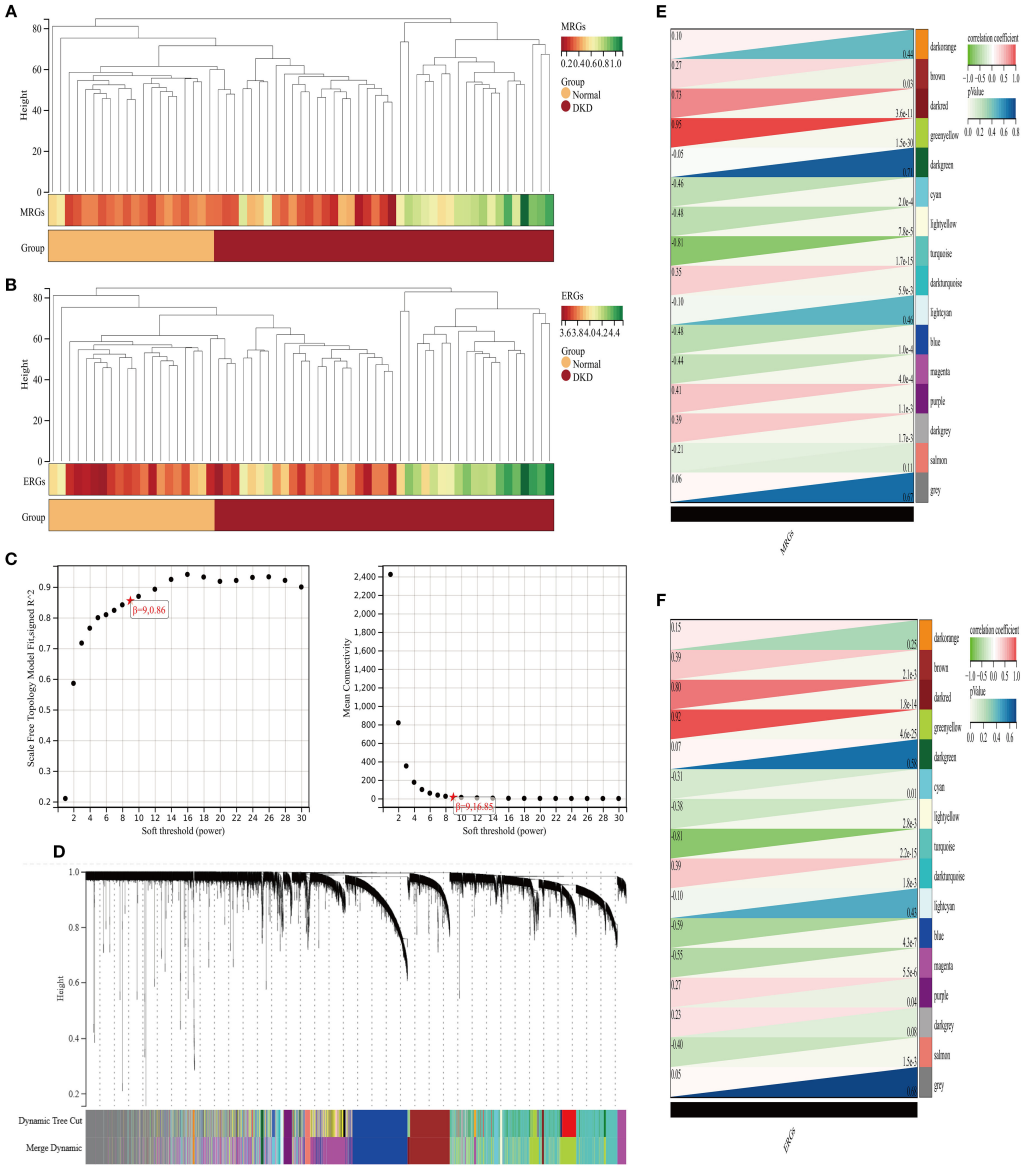
Analysis of gene modules associated with M2 macrophage polarization and efferocytosis in DKD. **(A, B)** Clustering analysis of characteristic genes **(A)** M2 macrophages; **(B)** efferocytosis; **(C)** Selection of the soft-thresholding power; **(D)** WGCNA dendrogram: Co-expression modules identified and color-coded, with a total of 16 characteristic modules; **(E, F)** Heatmap of correlations between co-expression modules **(E)** M2 macrophage polarization; **(F)** efferocytosis.

Enrichment analysis was performed using background gene sets constructed based on MRGs and ERGs, and no significant outliers were detected (**Figures 3A, B**). The optimal soft-thresholding power was identified as 9, based on the R² statistic and the mean connectivity criterion (**Figures 3C, D**). Hierarchical clustering analysis classified the gene expression profiles of GSE96804 dataset into 16 co-expression modules (**Figures 3E, F**). Among them, the green yellow module positively correlated with the M2 macrophage score (*r* = 0.95, *P* = 1.5×10^-30^), while the turquoise module negatively correlated (*r* = -0.81, *P* = 1.7×10^-15^) (**Figure 3E**). In efferocytosis-related characteristics, the green yellow (*r* = 0.92, *P* = 4.6×10^-25^) and dark red (*r* = 0.80, *P* = 1.80×10^-14^) modules exhibited significant positive correlations, whereas turquoise module negatively correlated (*r* = -0.81, *P* = 2.2×10^-15^) (**Figure 3F**). A total of 1,021 module genes associated with MRGs and 1,089 module genes associated with ERGs were ultimately identified.

### 3.3 Enrichment analysis of MEDEGs

**Figure 4:**
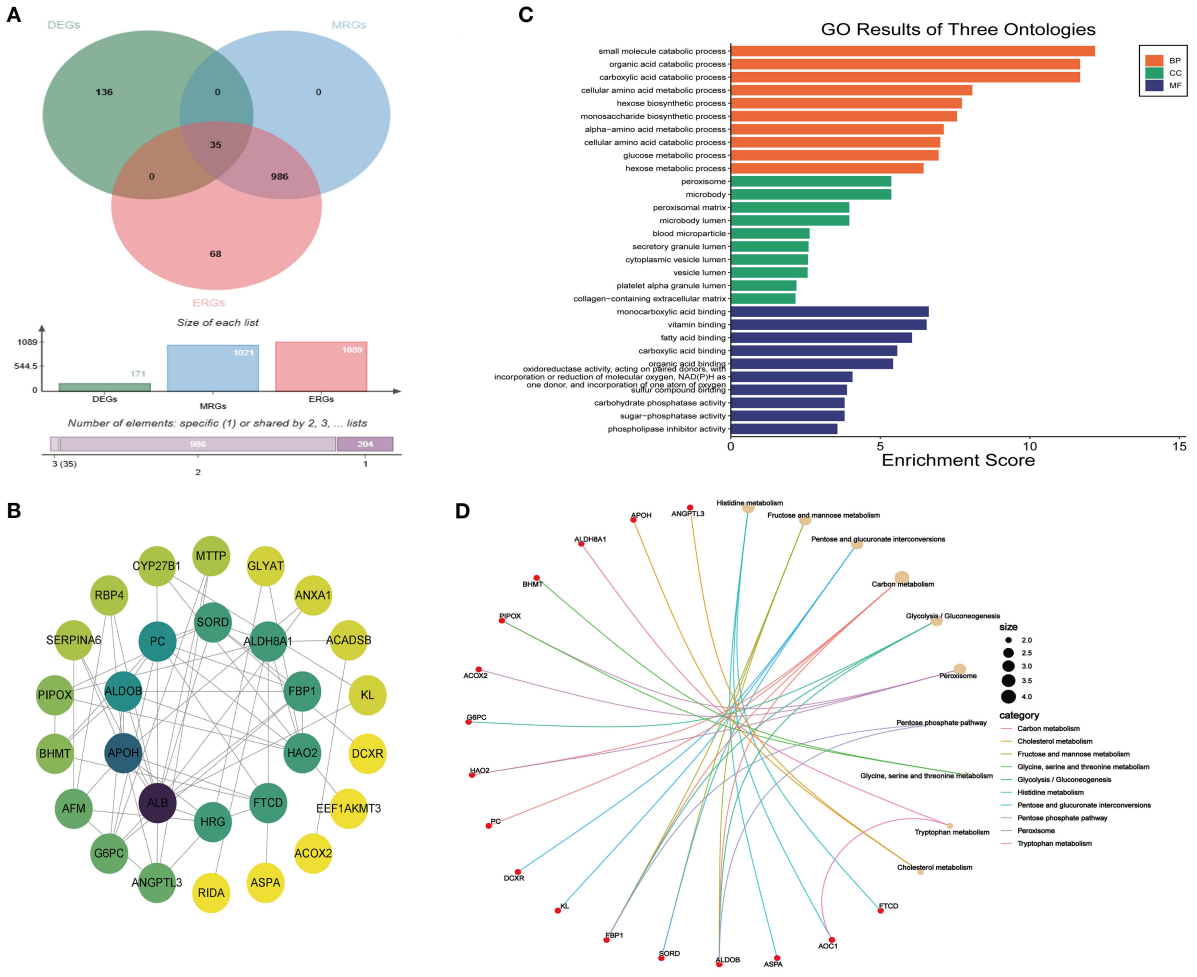
Enrichment analysis of MEDEGs. **(A)** Venn diagram of DEGs, ERGs, and MRGs.; **(B)** PPI network; **(C)** GO enrichment analysis; **(D)** KEGG enrichment analysis.

A total of 35 MEDEGs were identified (**Figure 4A**). The PPI network was constructed to analyze the interactions between the 35 MEDEGs (**Figure 4B**). Biological process (BP) was predominantly enriched in various metabolic processes, such as small molecule catabolism and organic acid degradation. The cellular component (CC) category was primarily enriched in microbody lumen, vesicle lumen, and secretory granule lumen. In the molecular function (MF) category, genes were enriched in binding to monocarboxylic acids, vitamins, and fatty acids (**Figure 4C**). KEGG pathway enrichment analysis were widely involved in various metabolic pathways, including carbon metabolism, pentose and glucuronate interconversions, and purine metabolism. The involvement of these pathways suggests that DEGs may play crucial roles in energy metabolism, substance conversion, and signal transduction (**Figure 4D**).

### 3.4 ML selection of hub genes

**Figure 5:**
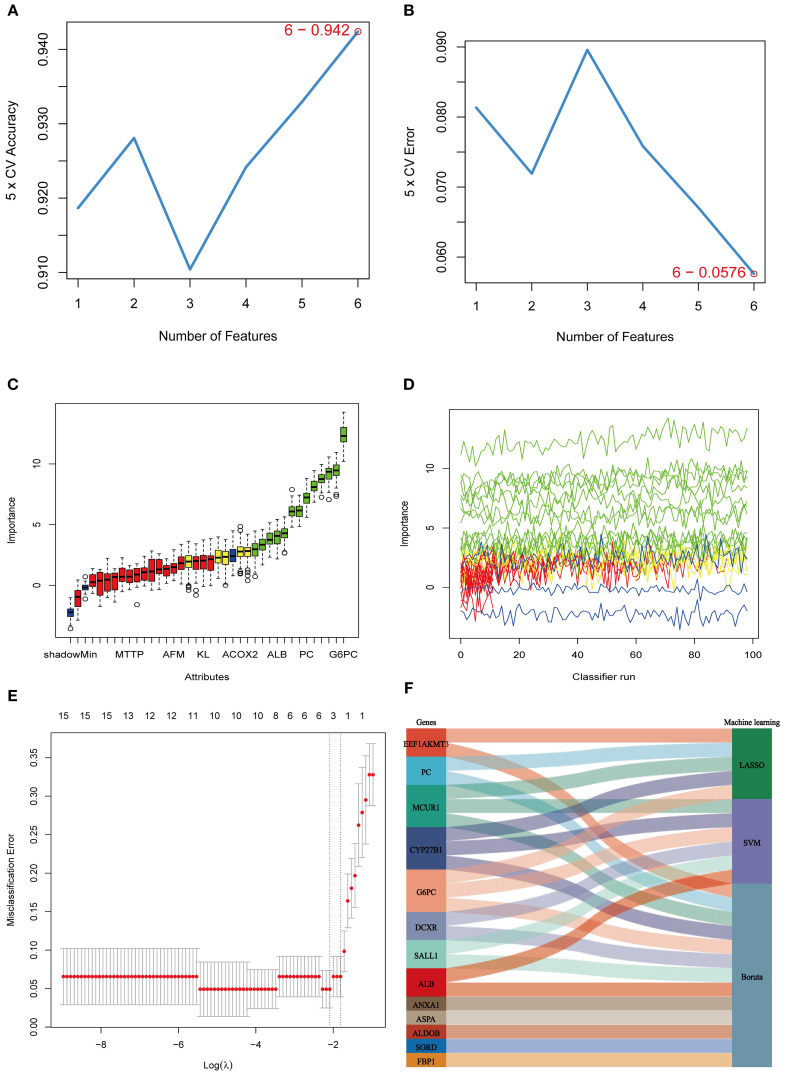
ML selection of hub genes. **(A)** Five-fold cross-validation accuracy of the SVM method under different feature numbers; **(B)** Five-fold cross-validation error of the SVM method under different feature numbers; **(C, D)** BORUTA algorithm results. **(E)** Cross-validation curve of LASSO regression; **(F)** Sankey diagram integrating the three machine learning approaches.

To further refine the selection of MEDEGs, three ML algorithms—SVM, BORUTA feature selection, and LASSO regression—were employed to screen hub genes (**Figure 5**). The SVM algorithm, utilizing a radial basis function kernel, identified six high-weight genes (**Figures 5A, B**). The BORUTA algorithm, based on random forest importance evaluation, identified 13 key genes (**Figures 5C, D**). LASSO regression, using ten-fold cross-validation, ultimately selected five feature genes (**Figure 5E**). A consensus analysis integrating all three algorithms identified MCUR1, CYP27B1, and G6PC as the core hub genes in DKD (**Figure 5F**).

### 3.5 Validation and clinical significance

**Figure 6:**
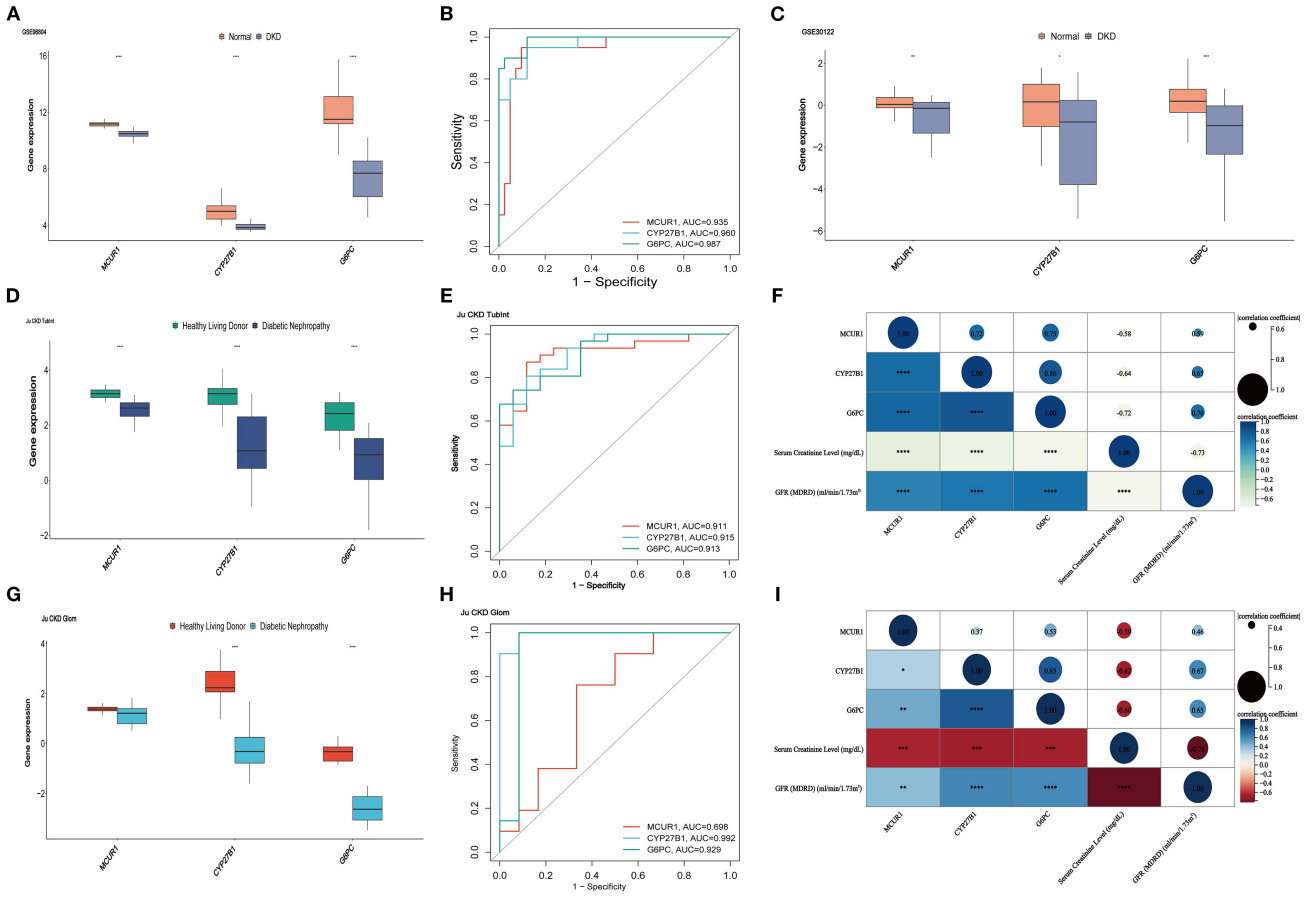
Validation and clinical correlation analysis. **(A)**hub genes in GSE96804 dataset; **(B)** ROC curves of hub genes in GSE96804; **(C)** hub genes in the GSE30122 dataset; **(D)** hub genes in the Ju CKD TubInt dataset; **(E)** ROC curves of hub genes in Ju CKD TubInt; **(F)** Correlation between hub genes and clinical parameters in Ju CKD TubInt; **(G)** hub genes in the Ju CKD Glom dataset; **(H)** ROC curves of hub genes in Ju CKD Glom; **(I)** Correlation between hub genes and clinical parameters in Ju CKD Glom (^*^ *P* < 0.05, ^**^ *P* < 0.01, ^***^ *P* < 0.001, ^****^ *P* < 0.0001).

The expression of MCUR1, CYP27B1, and G6PC were analyzed in the GSE96804 dataset, revealing significantly lower expression in the DKD group (*P* < 0.01) (**Figure 6A**). To further evaluate the predictive capability of these three genes in distinguishing DKD from normal samples, the AUC was calculated: MCUR1 (0.935), CYP27B1 (0.960), and G6PC (0.987) (**Figure 6B**). The expression trends of hub genes in the GSE30122 dataset, Ju CKD TubInt, and Ju CKD Glom were consistent with those observed in GSE96804 (**Figures 6C, D, G**). Except for MCUR1, which exhibited a lower AUC in Ju CKD Glom, all other AUC values exceeded 0.9 (**Figures 6E, H**), demonstrating strong discriminative capability and suggesting their potential value in the early diagnosis of DKD. **Figure 6F** and **Figure 6I** illustrate the correlation between MCUR1, CYP27B1, and G6PC and clinical parameters, including serum creatinine levels and GFR. Correlation analysis revealed a significant negative correlation between hub gene expression and Scr levels, while a positive correlation was observed with GFR. This suggests that alterations in these gene expressions are not only associated with DKD progression but may also play a crucial role in reflecting kidney function impairment.

### 3.6 Nomogram model

**Figure 7:**
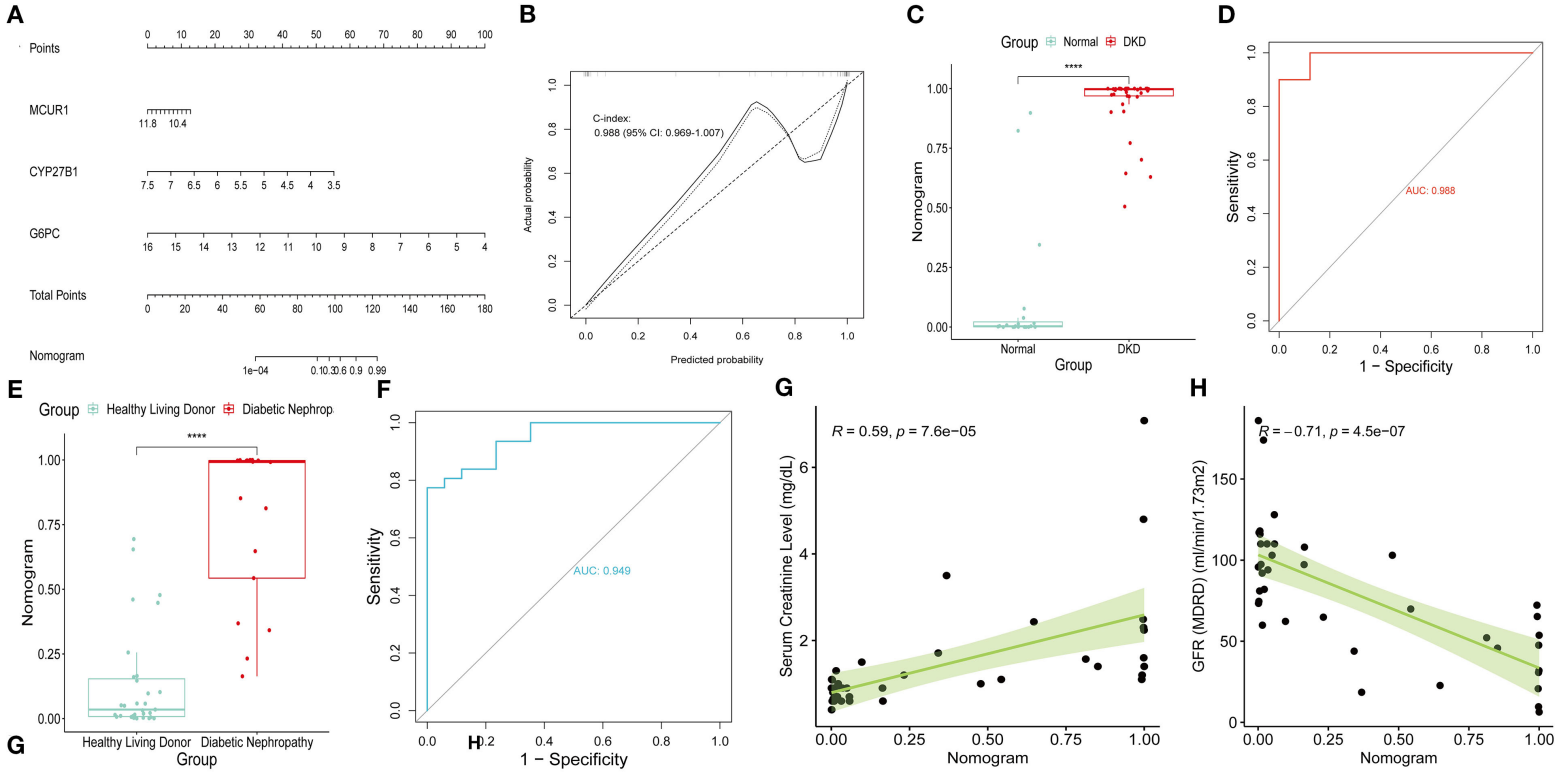
Nomogram model. **(A)** Nomogram; **(B)** Calibration curve; **(C)** Risk scores in GSE96804 dataset; **(D)** ROC curve of risk scores in GSE96804 dataset; **(E)** Risk scores in Ju CKD TubInt dataset; **(F)** ROC curve of risk scores in Ju CKD TubInt dataset; **(G)** Correlation between the risk scores and Scr; **(H)** Correlation between the risk scores and GFR.

A Nomogram model for predicting DKD was developed based on MCUR1, CYP27B1, and G6PC (**Figure 7A**). The calibration curve provided preliminary evidence of predictive accuracy for DKD (**Figure 7B**). In the GSE96804 dataset, the risk score was significantly higher in DKD group (*P* < 0.001) (**Figure 7C**). The AUC based on the risk score was 0.988, suggesting a promising discriminative ability (**Figure 7D**). Consistent results are also obtained in Ju CKD TubInt (AUC = 0.949) (**Figures 7E, F**). Notably, a positive correlation exists between risk score and Scr levels (*r* = 0.59, *P* < 0.001) and a negative correlation exists between risk score and GFR (*r* = -0.71, *P* < 0.001) (**Figures 7G, H**).

### 3.7 Immune infiltration analysis

**Figure 8:**
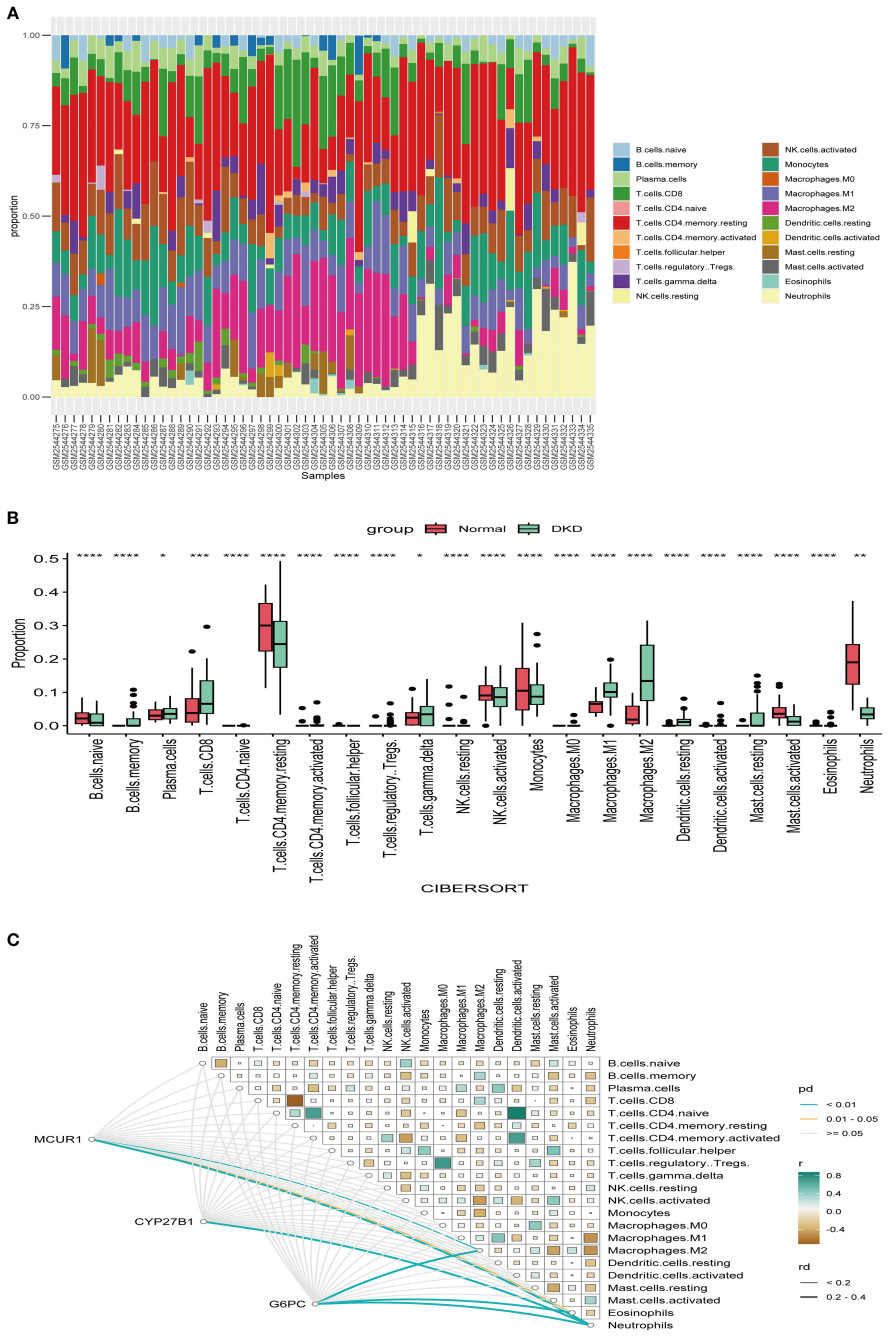
Immune infiltration. **(A)** Relative proportions of 22 immune cells; **(B)** Box plot comparing immune cell infiltration levels; **(C)** Heatmap illustrating the correlations between hub genes and immune cell types (^*^ *P* < 0.05, ^**^ *P* < 0.01, ^***^ *P* < 0.001, ^****^ *P* < 0.0001).

To explore the immune microenvironment of DKD, we employed the CIBERSORT algorithm to compare immune cell infiltration levels (**Figures 8A, B**). Several immune cell subsets, including γδ T cells, NK cells, and macrophages (M0, M1 and M2), were significantly elevated in DKD group. These findings indicate that immune cells could be crucial in the pathological progression of DKD. MCUR1 was positively correlated with M2 macrophages and neutrophils, CYP27B1 was associated with neutrophils, and G6PC exhibited correlations with M2 macrophages, neutrophils, and eosinophils (**Figure 8C**).

### 3.8 Sc-RNA seq analysis

**Figure 9:**
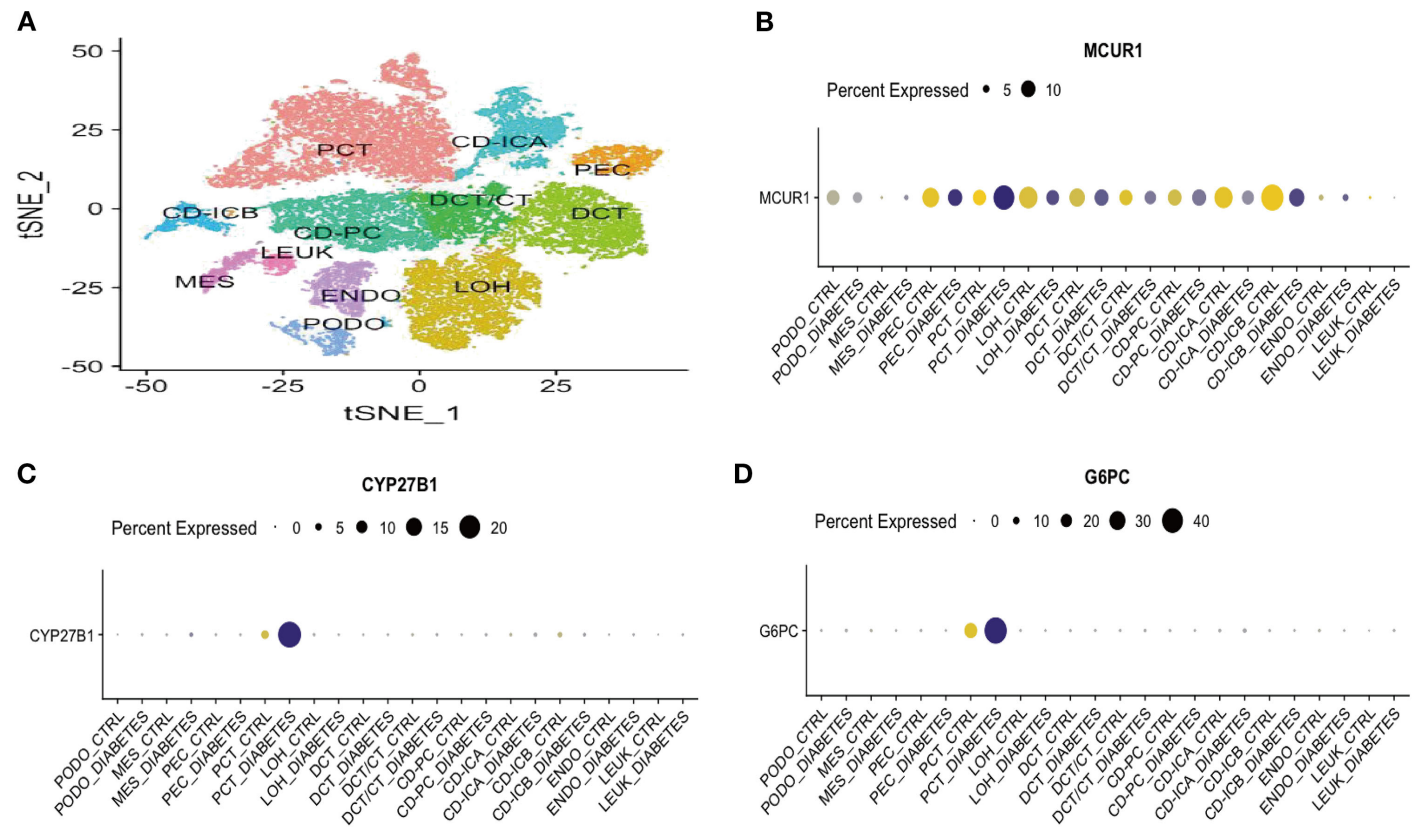
Sc-RNA seq expression analysis **(A)** t-SNE plot of single-cell sequencing data; **(B)** Bubble plot of MCUR1 expression levels; **(C)** Bubble plot of CYP27B1 expression levels; **(D)** Bubble plot of G6PC expression levels.

To gain deeper insights, we performed sc-RNA seq analysis in KIT database. **Figure 9A** displays the t-distributed stochastic neighbor embedding (t-SNE) plot of sc-RNA data in DKD. The analysis identified 12 distinct cell clusters, representing various renal cell types, including podocytes (PODO), proximal convoluted tubule cells (PCT), loop of Henle cells (LOH), endothelial cells (ENDO), and parietal epithelial cells (PEC). Regarding gene expression patterns, MCUR1 was primarily localized in PEC, PCT, and LOH. In contrast, CYP27B1 and G6PC were predominantly expressed in PCT (**Figures 9C, D**).

### 3.9 Consensus clustering

**Figure 10:**
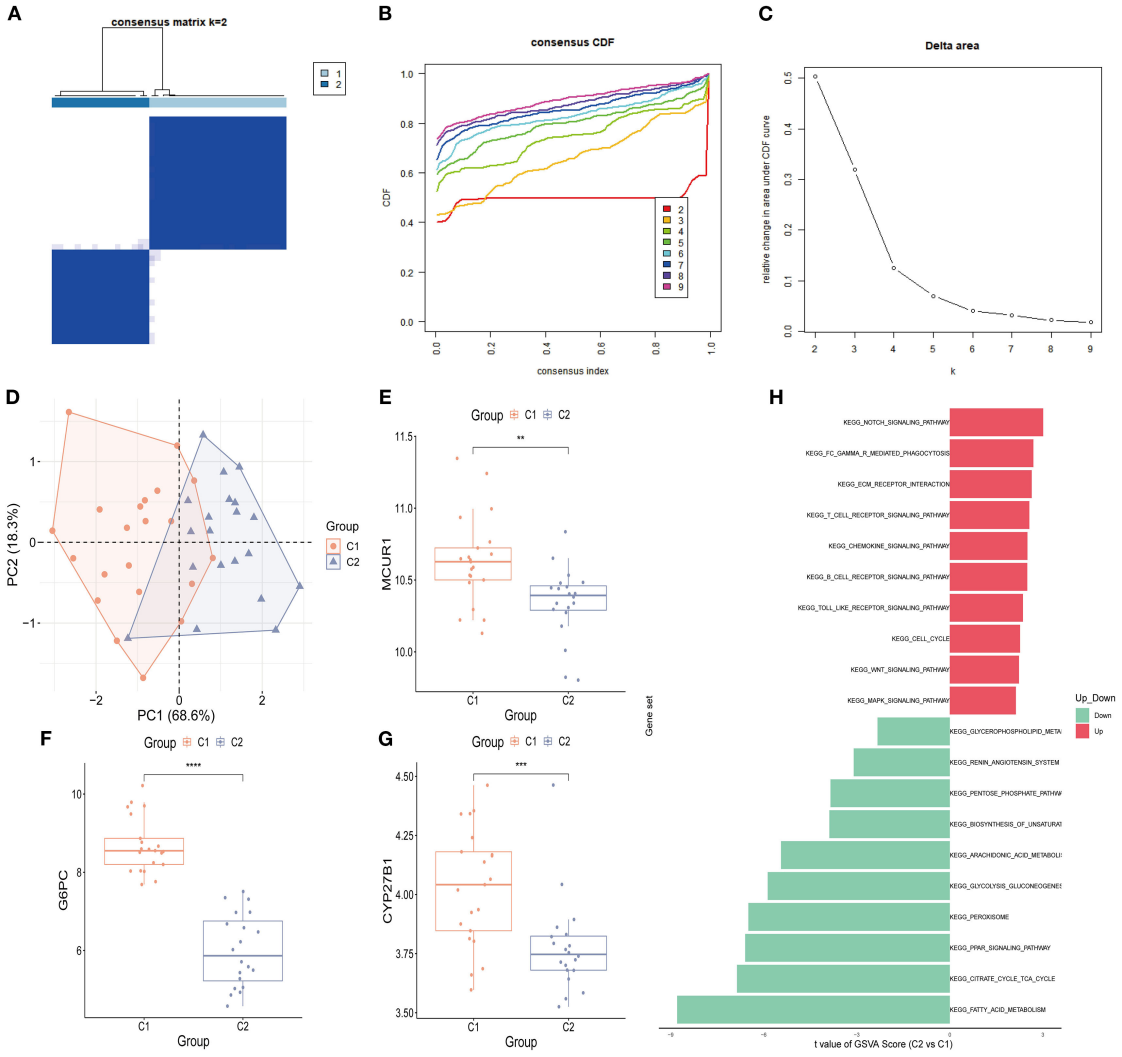
Consensus clustering analysis. **(A)** Consensus clustering matrix (k = 2); **(B)** CDF curve; **(C)** Area under the CDF curve; **(D)** PCA plot of two subtypes; **(E-G)** Box plots of hub genes expression (^*^ *P* < 0.05, ^**^ *P* < 0.01, ^***^ *P* < 0.001, ^****^ *P* < 0.0001).; **(H)** GSVA analysis between two subtypes.

Consensus clustering was conducted to classify 41 DKD patients into distinct molecular subtypes. By comprehensively evaluating cluster stability indicators, the optimal cluster number was determined as k = 2. The consensus matrix heatmap, cumulative distribution function (CDF) curve, and cluster stability analysis collectively confirmed the robustness of this clustering strategy (**Figures 10A-C**). As a result, 41 DKD patients were divided into two subgroups: C1 (*n* = 21) and C2 (*n* = 20). Further PCA demonstrated notable differences between two subtypes (**Figure 10D**). The box plots of hub gene expression levels clearly demonstrated that MCUR1, CYP27B1, and G6PC were significantly higher in C1 group (**Figures 10E-G**). To explore functional differences between the two subgroups, GSVA was conducted. The results showed that the Renin-Angiotensin System and Glycerophospholipid Metabolism pathways were upregulated in C1, whereas NOTCH signaling, Gamma R-mediated phagocytosis, ECM-receptor interactions, and chemokine signaling pathways were upregulated in C2 (**Figure 10H**).

### 3.10 Molecular regulatory network and drug prediction

**Figure 11:**
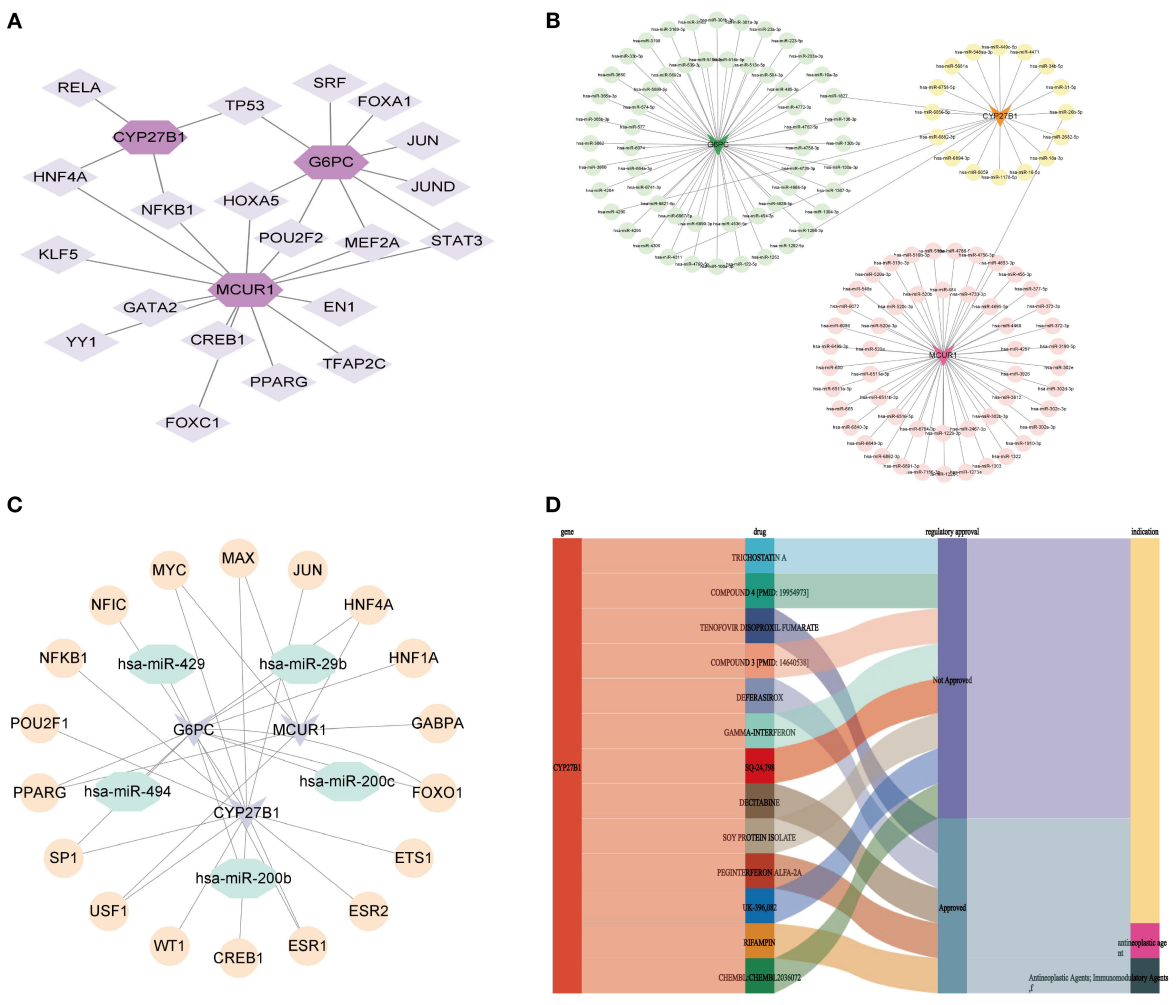
Molecular regulatory network and drug prediction. **(A)** mRNA-TF network; **(B)** mRNA-miRNA network; **(C)** TF-miRNA-mRNA network; **(D)** Drug prediction.

To elucidate the regulatory mechanisms of hub genes in DKD, a molecular regulatory network was constructed, followed by drug prediction analysis. At the transcriptional regulation level, a 23-node, 27-edge network was identified, including 20 key TFs. Among them, NFKB1 and HNF4A were identified as regulators of MCUR1 and CYP27B1, while POU2F2, MEF2A, and HOXA5 specifically regulated MCUR1 and G6PC. Additionally, TP53 was found to exert dual-target regulation over CYP27B1 and G6PC (**Figure 11A**). The mRNA-miRNA interaction network was generated, predicting 123 miRNAs, forming a 126-node, 125-edge network (**Figure 11B**). Subsequently, we constructed the TF-miRNA-mRNA network to provide a clearer and more intuitive depiction of the interrelationships among mRNA, miRNA, and TF (**Figure 11C**). Notably, drug prediction analysis using the DGIdb database revealed that CYP27B1 could serve as a potential therapeutic target for 13 drugs (**Figure 11C**).

### 3.11 Animal experiment validation

**Figure 12:**
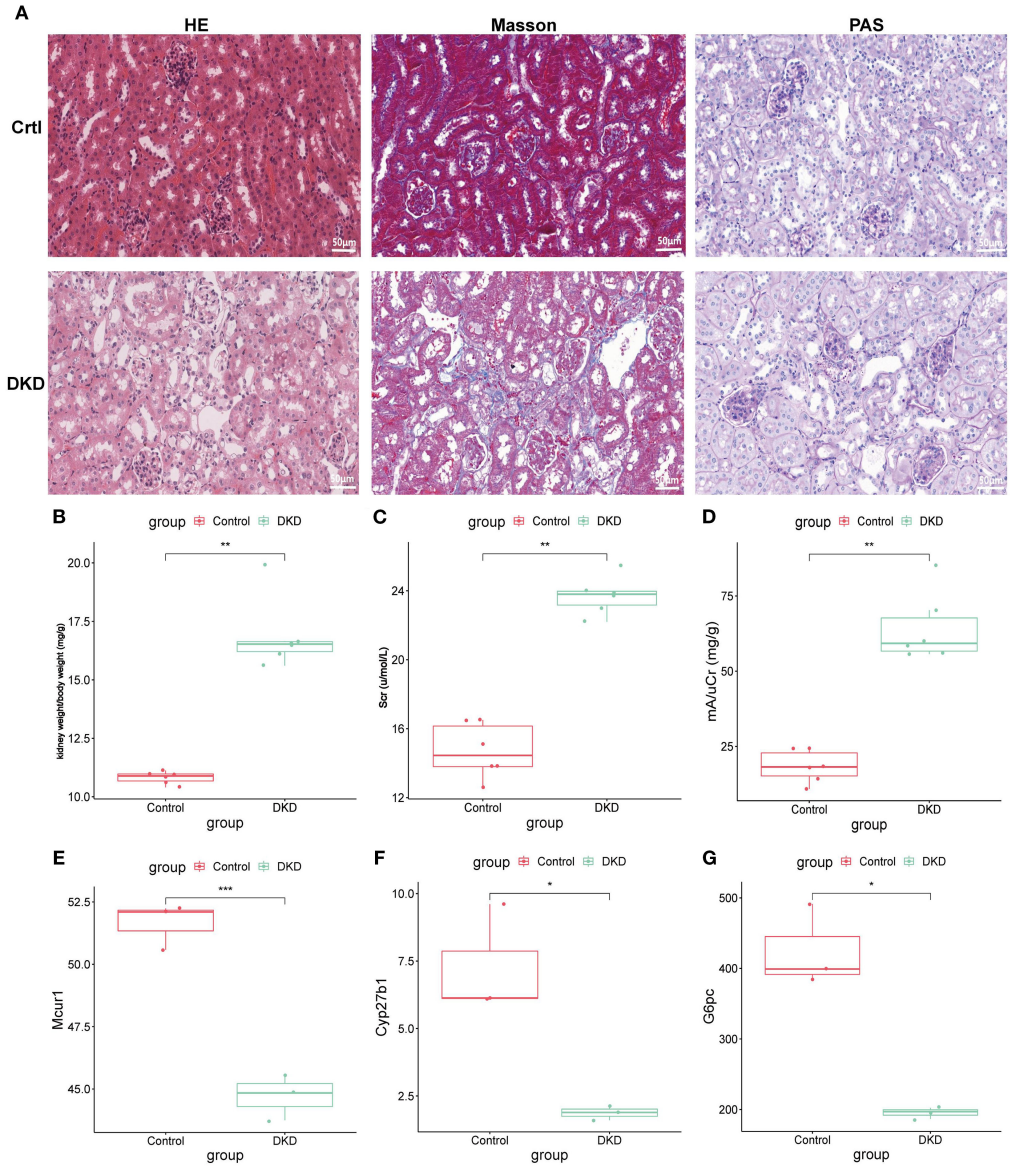
Animal experiment validation. **(A)** HE, PAS, and Masson staining (×400; 50μm). **(B)** kW/BW at 20 weeks. **(C)** Scr at 20 weeks. **(D)** mA/uCr at 20 weeks. **(E-G)** mRNA expression of Mcur1, Cyp27b1, and G6pc (^*^ *P* < 0.05, ^**^ *P* < 0.01, ^***^ *P* < 0.001).

Further exploration of hub genes expression was carried out using the DKD mouse model. Histological analysis (**Figure 12A**): HE, PAS, and Masson staining were used to compare the renal pathology. The control group showed normal glomerular morphology, well-arranged renal tubules, and no significant pathological alterations. The DKD group exhibited classic DKD pathological features, including glomerular hypertrophy, diffuse thickening of the tubular basement membrane, and extensive collagen deposition in the renal interstitium, indicating significant renal interstitial fibrosis. Renal function assessment (**Figures 12B-D**): The kidney-to-body weight ratio (kW/BW) was significantly increased in DKD group. Scr and urine microalbumin/creatinine ratio (mAlb/uCr) were both significantly elevated, confirming renal dysfunction in DKD mice. Transcriptomic validation (**Figures 12E-G**): Whole-transcriptome sequencing was performed to validate hub genes expression. Compared to control group, the mRNA expression levels of Mcur1, Cyp27b1, and G6pc in DKD renal tissues were significantly downregulated (*P* < 0.05).

## 4 Discussion

DKD pathogenesis involves complex metabolic disorders, inflammatory responses, and immune microenvironment dysregulation. This study systematically identified key genes related to M2 macrophage polarization and efferocytosis (MCUR1, CYP27B1, and G6PC) using multi-dataset analysis, machine learning, and animal-level transcriptomic sequencing, revealing their potential roles in immune-metabolic regulation in DKD and constructing a predictive model.

### 4.1 The pathological significance of M2 macrophage polarization and efferocytosis in DKD

Recent studies have highlighted that macrophage phenotype polarization (M1/M2 balance) and dysfunctional efferocytosis are critical factors in the progression of DKD (14, 30). Our study found that the M2 macrophage score was significantly elevated in DKD patients. M2 macrophages contribute to alleviating renal injury through their anti-inflammatory and tissue-repair functions. However, excessive M2 polarization is considered a risk factor for tissue fibrosis (31, 32). Additionally, efferocytosis, a key mechanism for clearing apoptotic cells, plays a central role in maintaining tissue homeostasis. Its dysfunction can lead to the accumulation of apoptotic debris, triggering secondary necrosis and inflammatory storms (33–35). Although some studies have shown that M1 macrophages possess phagocytic capability, the majority of research supports that M2 macrophages exhibit stronger efferocytosis compared to M1 macrophages (36–42). Interestingly, macrophage efferocytosis promotes polarization towards the M2 phenotype, a process regarded as essential for resolving inflammation and facilitating tissue repair, thereby aiding in the restoration of tissue homeostasis (37, 43–46). In this study, the elevated ERGs score in the DKD group may be associated with an increased demand for apoptotic cell clearance; however, whether its efficiency is impaired requires further validation.

### 4.2 Regulatory network and functional analysis of hub genes

MCUR1 is a key regulatory protein of the MCU complex, primarily expressed in the inner mitochondrial membrane. It modulates MCU complex activity, regulating mitochondrial calcium uptake, thereby influencing cellular energy metabolism and apoptosis (47). Efferocytosis is a mitochondria-mediated process, where mitochondrial metabolism, dynamics, and inter-organelle communication influence the clearance of apoptotic cells by phagocytes (48, 49) and are critical for macrophage activation and differentiation (50). In an atherosclerotic mouse model, MCUR1 was found to regulate macrophage endocytosis influenced by oxidized low-density lipoprotein (oxLDL) (51). However, no studies have yet investigated MCUR1 in DKD, highlighting the novelty of our findings.

CYP27B1 is a cytochrome P450 enzyme primarily involved in vitamin D (VD) metabolism (52). Besides regulating calcium and bone homeostasis, VD plays a crucial role in immune system, particularly in modulating macrophage polarization and function (53, 54). In DKD studies, active VD has been shown *in vitro* to promote the transition of high-glucose-induced M1 macrophages to the M2 phenotype, reducing inflammation to prevent podocyte injury (55). Additionally, it can inhibit macrophage polarization towards the M1 phenotype via the STAT-1/TREM-1 pathway (56), thereby alleviating renal inflammation and fibrosis and ultimately preserving kidney function. Macrophages can synthesize active VD and express the VD receptor (VDR) in their nuclei. Their polarization state significantly affects their metabolic capacity for 25(OH)D3, with M2a macrophages exhibiting higher activity in synthesizing active VD, whereas M1 macrophages display lower activity (57). In CYP27B1 gene knockout mouse models, excessive 25(OH)D3 significantly exacerbated renal tubulointerstitial fibrosis and oxidative stress, pathological changes that are closely associated with macrophage phenotype alterations (58). VD can upregulate ASAP2 transcription, promoting efferocytosis by modulating cytoskeletal rearrangement and vesicular transport (59).

The G6PC gene encodes the catalytic subunit of glucose-6-phosphatase, playing a crucial role in glucose production and release, which is essential for maintaining glucose homeostasis (60). In liver-specific G6pc knockout mice, proteins associated with tissue inflammation and macrophage M2 polarization (such as ARG1 and CD163) were significantly upregulated (61). Renal G6pc deficiency leads to glucose-6-phosphate (G6P) accumulation, which activates the pentose phosphate pathway and *de novo* lipogenesis, resulting in triglyceride accumulation. The activation of the renin-angiotensin system and increased TGF-β1 levels contribute to epithelial-mesenchymal transition (EMT) and podocyte injury, ultimately impairing the glomerular filtration barrier (62). However, research on the role of G6PC in macrophages and DKD remains unexplored.

### 4.3 Molecular subtyping and potential applications in precision medicine

Consensus clustering analysis classified DKD patients into two subtypes, C1 and C2, based on hub genes. The C1 subtype is characterized by activation of the RAS pathway and glycerophospholipid metabolism, while the C2 subtype is enriched in the NOTCH signaling and extracellular matrix (ECM) remodeling pathways. This classification suggests that: (1) C1 subtype patients may benefit from a combination of RAS inhibitors (e.g., ACEI/ARB) and lipid metabolism modulators; (2) C2 subtype patients may require targeted interventions against fibrosis-related pathways, such as TGF-β and ECM receptors. Additionally, the higher expression of hub genes in the C1 subtype may indicate stronger renal compensatory capacity, suggesting a better prognosis compared to the C2 subtype. This molecular subtyping provides new insights for individualized treatment strategies in DKD.

### 4.4 Immune microenvironment imbalance: from mechanisms to therapeutic targets

Immune infiltration analysis revealed significantly increased levels of M0, M1, and M2 macrophages, CD8+ T cells, and NK cells in the DKD group, suggesting that both the innate and adaptive immune systems contribute to the pathogenesis of DKD. The abnormal infiltration of these immune cells may exacerbate inflammatory responses and worsen tissue damage. Notably, the increased infiltration of M2 macrophages in DKD may reflect the body’s attempt to counteract inflammation through their anti-inflammatory effects. Moreover, M2 macrophages exhibit a correlation with hub genes, indicating that these genes may regulate macrophage metabolic phenotypes and influence their functions. For instance, MCUR1-mediated mitochondrial calcium signaling may modulate macrophage cytokine secretion, while G6PC influences macrophage polarization through glucose metabolism. Targeting the immune-metabolic microenvironment, such as modulating the glycolysis/oxidative phosphorylation balance in macrophages, may offer a potential new therapeutic approach for DKD.

Sc-RNA seq analysis further revealed the distribution patterns of MCUR1, CYP27B1, and G6PC across different renal cell types. These genes are primarily expressed in parietal epithelial cells of the glomerulus, proximal convoluted tubule cells, and proximal tubular cells, all of which play crucial roles in the pathogenesis of DKD. For example, damage to parietal epithelial cells is closely linked to the integrity of the glomerular filtration barrier, while injury to proximal convoluted and proximal tubular cells may lead to tubular dysfunction and proteinuria. Therefore, the downregulation of these genes in specific cell types may directly impact renal structure and function. Animal experiments confirmed the downregulation of MCUR1, CYP27B1, and G6PC in DKD mouse models, further supporting their potential diagnostic value in DKD.

### 4.5 Limitations

This study has the following limitations: (1) This study is based on public databases; however, further *in vitro* and *in vivo* functional studies, such as gene knock-out or overexpression, are required to validate the role of hub genes in efferocytosis and macrophage polarization; (2) The study primarily focuses on the roles of MCUR1, CYP27B1, and G6PC in macrophages. However, the mechanisms by which these genes interact with renal tubular and glomerular cells remain unclear. Furthermore, the specific role of these interactions in the pathogenesis of DKD is not well understood. (3) Although the critical roles of MCUR1, CYP27B1, and G6PC in M2 macrophage polarization and macrophage apoptosis phagocytosis in DKD have been revealed, the specific molecular pathways through which these key genes regulate M2 macrophage polarization and apoptosis phagocytosis remain unclear. Future research should focus on further validating, through *in vitro* and *in vivo* experiments, the role of hub genes in phagocytosis or efferocytosis polarization. Additionally, it should investigate the molecular mechanisms underlying the regulation of macrophage-tubule/glomerular cell interactions by hub genes, while optimizing models based on these key genes. Furthermore, the feasibility of using these models as non-invasive diagnostic tools or therapeutic monitoring biomarkers should be explored.

## 5 Conclusion

This study systematically identified MCUR1, CYP27B1, and G6PC as key genes associated with M2 macrophage polarization and efferocytosis in DKD through multi-dataset analysis, machine learning, and experimental validation. They may contribute to DKD development via metabolic and immune pathways. Future research should investigate their regulatory mechanisms and evaluate their potential as therapeutic targets.

## Data Availability

The datasets used and/or analysed during the current study are available from the corresponding author on reasonable request.
